# The Past, Present, and Future of Health Sciences Research

**DOI:** 10.1093/geroni/igaa057.2915

**Published:** 2020-12-16

**Authors:** Cynthia Brown, John Batsis

## Abstract

The health sciences have experienced an evolution in research over the past 75 years, moving along the translational spectrum from bench to bedside to the community. This transformation has impacted health outcomes for older adults at a global and public health level. Expansion in geroscience and implementation science research has changed the lens through which we view how aging occurs, precision management of disease, and has allowed the integration of tested interventions into healthcare systems. This Presidential symposium will showcase investigators, ranging from junior to senior researchers, who will share where their science began, how their research has built on the past, provide insights into their own work, and share their perspectives on the continuing trajectory of their scientific work.

